# Supplementary material for: Acute Gaseous Air Pollution Exposure and Hospitalizations for Acute Ischemic Stroke: A Time-Series Analysis in Tianjin, China
Source: Int J Environ Res Public Health. 2022 Oct 16;19(20):13344. doi: 10.3390/ijerph192013344 (PMC9603069; doi:10.3390/ijerph192013344)
Supplement: Supplementary file 1 [file ijerph-19-13344-s001.zip › ijerph-1873296-supplementary.pdf]

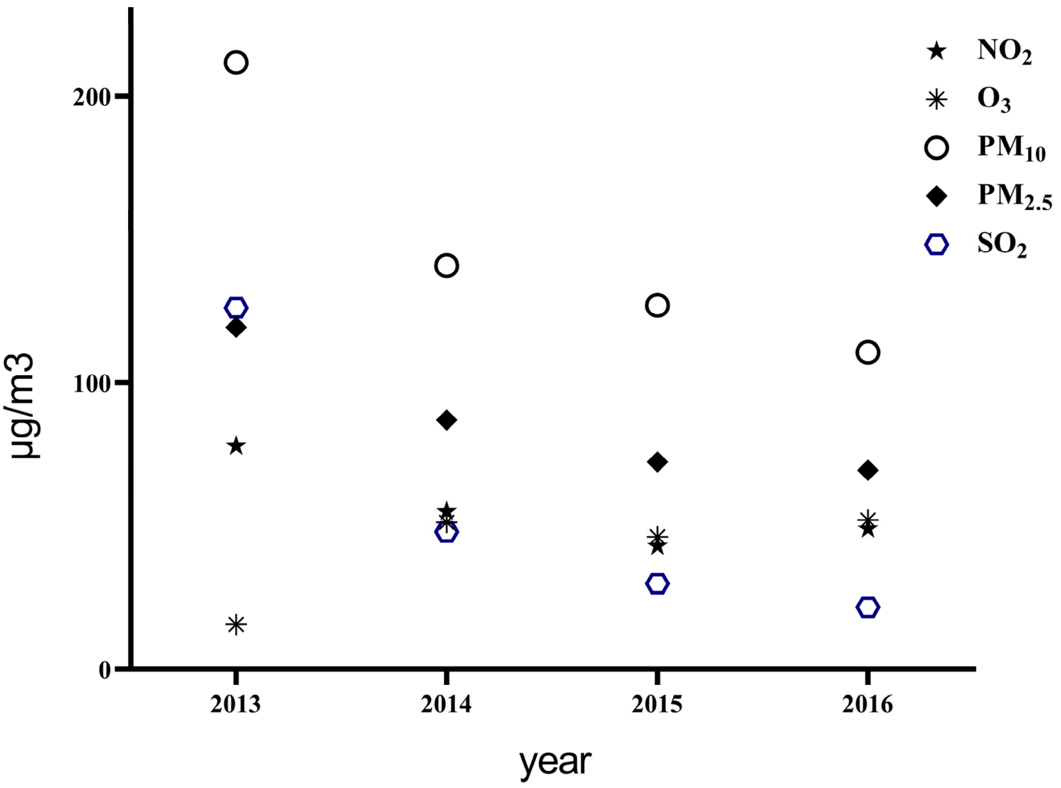

Figure S1 Time series plot of NO2, SO2, PM2.5, PM10, and O3

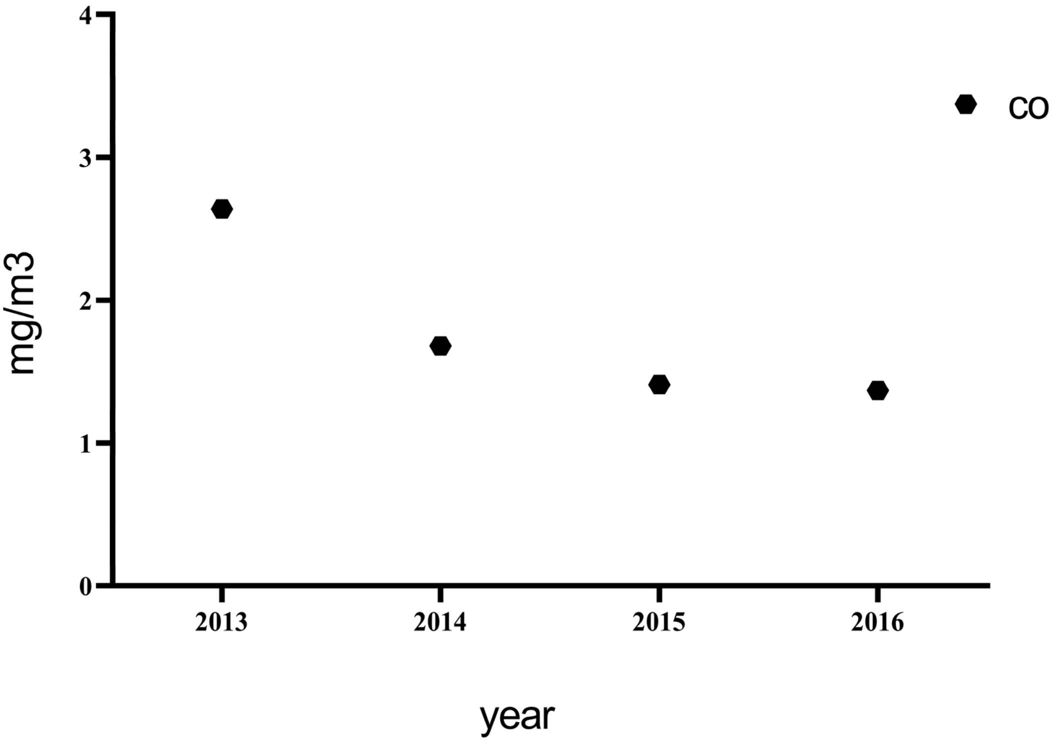

Figure S2 Time series plot of CO

Table S1 Percentage change with 95%CI of daily hospitalizations for ischemic stroke with 10 μg/m³ or 10 mg/m³ increase in gaseous pollutant concentration in single-day lag analysis in Tianjin (2013－2016)

Table Percentage change with 95%CI of daily hospitalizations for ischemic stroke with 10 μg/m³ or 10 mg/m³ increase in gaseous pollutant concentration in single-day lag analysis in Tianjin (2013－2016)

Lag	NO_2	SO_2	CO	O_3
Lag0	1.007(0.995-1.020)	1.004(0.994-1.014)	1.178(0.851-1.630)	1.004(0.997-1.011)
Lag1	0.997(0.985-1.010)	1.004(0.994-1.014)	1.130(0.816-1.565)	1.006(0.999-1.013)
Lag2	0.998(0.986-1.010)	0.999(0.989-1.009)	1.067(0.770-1.479)	0.999(0.992-1.006)
Lag3	0.985(0.973-0.997)*	0.995(0.985-1.005)	0.887(0.637-1.234)	1.003(0.996-1.010)
Lag4	0.998(0.986-1.010)	1.000(0.990-1.010)	1.087(0.781-1.512)	0.994(0.987-1.001)
Lag5	0.996(0.984-1.008)	0.996(0.986-1.007)	0.912(0.655-1.269)	0.997(0.990-1.004)
Lag6	0.992(0.980-1.005)	0.997(0.987-1.007)	0.761(0.545-1.063)	0.999(0.992-1.006)
Lag7	0.987(0.974-0.999)*	0.987(0.977-0.998)*	0.747(0.536-1.041)	1.002(0.995-1.009)

Note: *$P < 0.05$

Table S2 Percentage change with 95%CI of daily hospitalizations for ischemic stroke with 10 μg/m3 or 10 mg/m3 increase in gaseous pollutant concentration in sliding average lag model lag analysis in Tianjin (2013—2016)

Table Percentage change with 95%CI of daily hospitalizations for ischemic stroke with 10 μg/m3 or 10 mg/m3 increase in gaseous pollutant concentration in sliding average lag model lag analysis in Tianjin (2013—2016)

Lag	NO_2	SO_2	CO	O_3
Lag1	1.004(0.989-1.018)	1.007(0.994-1.019)	1.224(0.839-1.788)	1.007(0.999-1.015)
Lag2	1.003(0.987-1.020)	1.008(0.993-1.023)	1.280(0.831-1.971)	1.005(0.996-1.014)
Lag3	0.994(0.976-1.013)	1.004(0.988-1.022)	1.182(0.727-1.922)	1.006(0.996-1.016)
Lag4	0.992(0.973-1.013)	1.002(0.983-1.021)	1.186(0.692-2.033)	1.003(0.992-1.014)
Lag5	0.990(0.969-1.012)	0.999(0.978-1.020)	1.119(0.618-2.027)	1.002(0.990-1.014)
Lag6	0.986(0.964-1.009)	0.996(0.973-1.019)	0.947(0.496-1.810)	1.001(0.988-1.015)
Lag7	0.980(0.956-1.005)	0.987(0.963-1.012)	0.811(0.404-1.628)	1.002(0.988-1.017)

Note: *P < 0.05
